# Cultivating inclusive instructional and research environments in ecology and evolutionary science

**DOI:** 10.1002/ece3.7062

**Published:** 2020-12-07

**Authors:** Nathan C. Emery, Ellen K. Bledsoe, Andrew O. Hasley, Carrie Diaz Eaton

**Affiliations:** ^1^ Department of Plant Biology Michigan State University East Lansing MI USA; ^2^ Department of Biology University of Regina Regina SK Canada; ^3^ BioQUEST Curriculum Consortium Boyds MD USA; ^4^ Digital and Computational Studies Bates College Lewiston ME USA

**Keywords:** diversity, equity, Inclusivity, research, teaching

## Abstract

As we strive to lift up a diversity of voices in science, it is important for ecologists, evolutionary scientists, and educators to foster inclusive environments in their research and teaching. Academics in science often lack exposure to research on best practices in diversity, equity, and inclusion and may not know where to start to make scientific environments more welcoming and inclusive. We propose that by approaching research and teaching with empathy, flexibility, and a growth mind‐set, scientists can be more supportive and inclusive of their colleagues and students. This paper provides guidance, explores strategies, and directs scientists to resources to better cultivate an inclusive environment in three common settings: the classroom, the research laboratory, and the field. As ecologists and evolutionary scientists, we have an opportunity to adapt our teaching and research practices in order to foster an inclusive educational ecosystem for students and colleagues alike.

## Graphical abstract

Inclusivity is critical for a scientifically informed future that reflects the diverse world that benefits from ecological and evolutionary inquiry. We propose that by approaching research and teaching with empathy, flexibility, and a growth mind‐set, scientists can be more supportive and inclusive of their colleagues and students. This paper provides guidance, explores strategies, and directs scientists to resources to better cultivate inclusivity in three common environments: the classroom, the research laboratory, and the field.

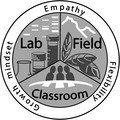

## 1 INTRODUCTION

Inclusivity is critical for a scientifically informed future that reflects a diverse world and benefits from ecological and evolutionary inquiry. Inclusivity overlaps with diversity and equity in that to truly include a broad diversity of people in science, there must be equitable opportunities in research and the classroom, providing a welcoming and inclusive environment for diverse ideas and perspectives to flourish. While higher education continues to push for greater diversity, equity, and inclusion (Smith, 2009), ecology and evolution as disciplines have historically not been welcoming for all people (O'Brien et al., 2020; Wanelik et al., 2020). Ecology and environmental organizations have not been open to diversity and inclusion in the past (Lawrence et al., 1993; Melosi, 1995; Taylor, 2007), but some progress has been made (Beck et al., 2014; Ortega et al., 2006). Evolutionary science is entangled with eugenics (Bashford & Levine, 2010) and race science (Jackson & Weidman, 2006) in ways that manifest even today (Daar, 2017). Scientists and educators have the power to shift ecology and evolution in a positive direction and build a more inclusive environment for future generations. The following article is meant to contribute to the ongoing conversation and propose some guidance to ecologists and evolutionary scientists by describing and providing *research‐based practices* to implement in everyday teaching and research settings with ample *citations to research articles for further reading*.

### 1.1 Positionality Statement

We draw from the education and social science literature, our personal experiences as scientists and educators, and conversations with colleagues, students, and organizations interested in making science and science education more inclusive. This paper is the product of a yearlong journey together to synthesize practices from inclusive pedagogy (Dewsbury & Brame, 2019) and Universal Design for Learning (UDL; Meyer et al., 2014) and then apply this synthesized framework to life science education and research mentoring. It is often the case that the silos of academia contribute to the silos of our conversations on equity and inclusion. Our goal was to leverage our different lived experience and expertise as well as our common passion for science and science education into a *shared framework for reflection*.

While three of the authors self‐identify as members of some underserved groups (i.e., women, the queer community, blind, Latinx), we are aware that we (a) do not speak for all members of the communities to which we belong and (b) do not represent all axes of diversity. We acknowledge our privilege and power as white, educated individuals in the academy. We recognize that we cannot fully understand the experiences of all scientists; we do, however, strive to be accomplices, co‐conspirators, and allies to and with marginalized and underserved groups in science through meaningful action to promote inclusivity. As Jackson et al. said in their book # HashtagActivism (2020), “In its most useful and radical form, allyship then draws from the idea that no one can be free unless everyone is free” (Collective, 1983; for more on “allyship,” see Appendix S1A). As such, we seek to encourage self‐reflection and collaboration and to nurture an ongoing dialogue about issues of inclusion and drive a more intersectional approach in the design of educational and professional spaces (see Definitions). Those who are the most underserved in the academy are those who have multiple marginalized identities. As such, it is important to adopt multiple instructional and research practices that directly prioritize their well‐being in the academy.

### 1.2 Development

Through our mutual interest in inclusive education, we were brought together as part of the inaugural Open Education Community Fellows program, a joint effort of the Environmental Data Science Inclusion Network (EDSIN, https://qubeshub.org/community/groups/edsin; Lauer et al., 2020) and Quantitative Undergraduate Biology Education and Synthesis (https://qubeshub.org; Akman et al., 2020; Donovan et al., 2015). Recognizing the need for a central community geared toward inclusive scientific (specifically biological and environmental) education, the EDSIN‐QUBES Open Education Community Fellows developed Biological, Universal, and Inclusive Learning in Data Science (BuiLDS), a site for collecting and sharing inclusive educational resources and creating a community of practice for inclusive education (see BuiLDS and additional useful resources in Appendix S1B).

As the group name acknowledges, there is substantial overlap between inclusive practices and UDL. Inclusive teaching practices, such as those summarized by Dewsbury and Brame (2019), originate primarily from creating educational experiences rooted in a racial justice perspective (Dewsbury, 2017). UDL, first outlined by CAST and intended for students with disabilities (www.cast.org), has its roots in Universal Design principals in architecture and recognizes that barriers to learning lie in design of the learning environment, not the individual learner. It provides an instructional perspective and framework that guides development of equitable learning experiences for the broadest possible diversity of students, minimizing the need for individual accommodations. We encourage readers to explore UDL and its role in fostering inclusivity using the resources provided in Appendix S1C.

In addition to the standard review process by Ecology and Evolution, this paper has undergone informal reviews from multiple colleagues invested in inclusivity issues in the biological sciences. This includes providing the EDSIN‐QUBES Open Education Community Fellows and their mentors the opportunity to read and comment on the manuscript. These efforts were made to improve and hone our message and provide opportunities for a multitude of voices to critique and leverage their expertise with respect to inclusivity in ecology and evolution.

The authors fully acknowledge that truly inclusive scientific and instructional environments require structural changes to the preexisting academic and research system (Danowitz & Tuitt, 2011; Hurtado et al., 1999, 2012; Mitchneck et al., 2016; Puritty et al., 2017; Vera et al., 2016; Winkle‐Wagner & Locks, 2013), as systemic racism, ableism, bigotry, and other prejudice pervade academia (Arday & Mirza, 2017; Dolmage, 2017; Harper, 2012; Museus et al., 2015). While some scientists and educators are positioned to enact such changes—and we strongly encourage them to do so—we also believe that widespread changes to research and teaching, enacted by scientists across disciplines, can have a positive impact. This article is meant as a starting point for ecological and evolutionary scientists and educators, as many of us are in a unique position to affect change through our roles as mentors, teachers, and principle investigators (Killpack & Melón, 2016; Macdonald et al., 2019; National Academies of Sciences, Engineering, & Medicine, 2019). Many small drops in a big pond can bring about a wave of change.

**Definitions: This is how we are using the following terms in this paper**

*Inclusivity*—“The practice of including people across differences. Inclusivity implies an intentional practice of recognizing and working to mitigate biases that lead to marginalization or exclusion of some people.” (Dewsbury & Brame, 2019)

*Diversity*—In higher education, there is structural diversity, the numerical representation of diverse groups (Hurtado et al., 1999), informal interactional diversity, or “the frequency and the quality of intergroup interaction as keys to meaningful diversity experiences during college,” and classroom diversity, where students are “learning about diverse people [content knowledge] and gaining experience with diverse peers in the classroom” (Gurin et al., 2002)

*Equity*—“Equality of opportunity…it is necessary to go beyond formal equality of rights and take account of differences in the opportunity structure.” (Clancy & Goastellec, 2007)

*Privilege*—“automatic unearned benefits bestowed upon perceived members of dominant groups based on social identity” (Case, 2013).

*Power*—“the ability to influence others to believe, behave, or to value as those in power desire them to” (French & Raven, 1959 in Mandelli, 2004)

*Intersectionality*—“axes of inequality pertaining to gender, race, and class that *intersect* with one another, i.e., that are interlocked, dependent upon one another, and mutually constituted.” (Veenstra, 2011) with origins in Black Feminism (see Crenshaw, 1989).

## 2 FRAMING YOUR RESEARCH AND TEACHING MIND‐SET

In our ecological and evolutionary research, we often encounter variation and adapt our approaches to better our science. Similarly, we suggest developing a mind‐set in your teaching and research that is adaptable to a diverse population (Burnette et al., 2013). This includes empathy, flexibility, and a growth mind‐set. Focusing on these three principles when designing and conducting your research and teaching will help you engage in practices that cultivate an inclusive environment in the classroom, in the laboratory, and in the field.

### 2.1 Empathy

While empathy is well established to have positive benefits in medical practice (Derksen et al., 2013), it is also important for interacting with students, mentees, and colleagues who are different from you (Bernier et al., 2005; Cole, 2008; Correia & Navarrete, 2017; Stephan & Finlay, 1999; Zembylas, 2012). Reflecting on our own privilege and empathizing with others' challenges and obstacles is one of many first steps to building a truly inclusive scientific environment. For example, first‐generation college students may be less familiar with institutional structures, policies, and culture than someone whose parents attended college (McCarron & Inkelas, 2006), and thus, first‐generation students may feel less comfortable engaging directly with faculty and classmates (Soria & Stebleton, 2012). By empathizing with students' hardships and reaching out to help, you, as a mentor, can help guide first‐generation students to be successful in academia. One helpful exercise for any scientist is to be aware of our own implicit bias; you can do so by participating in self‐guided exercises (e.g., Harvard implicit bias test) or implicit bias training (e.g., Kirwan Institute implicit bias training). Incorporating empathy into your teaching and research is not accomplished overnight and necessitates reflection, as empathy is susceptible to bias that can render it counterproductive (Prinz, 2011). It is a lifelong process of developing cultural humility, a commitment to self‐evaluation, self‐critique, and forming mutually beneficial relationships with students and peers (Tervalon & Murray‐Garcia, 1998).

### 2.2 Flexibility

Just as we are flexible in our approaches to scientific investigations, maintaining flexibility with your peers and students is also important. Students—graduate and undergraduate—experience numerous difficulties and obstacles that may be unknown or unfamiliar to colleagues and mentors. Some students, for example, may have obligations and responsibilities that are obscure to faculty and mentors (MacDonald, 2018). To address some of these complexities, mentors can, for instance, be flexible in scheduling meetings with students who may not be able to adhere to a rigid weekly schedule. Additionally, listening to student concerns and incorporating student feedback into research and curricular design may seem obvious and simple, but open educational practices can have tremendous positive impacts on students and mentees (Bali et al., 2020; Carey et al., 2015). Open communication with peers and students and incorporating flexibility into research and teaching design will contribute significantly to fostering an inclusive environment (Barnett, 2013).

### 2.3 Growth mind‐set

**FIGURE 1:**
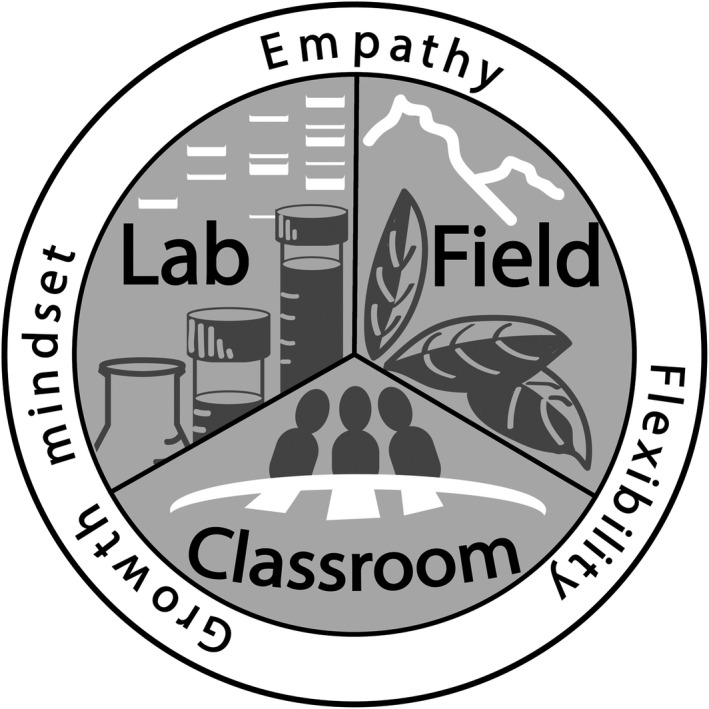
The three principles of empathy, flexibility, and a growth mind‐set will help ecologists and evolutionary scientists promote inclusivity in the classroom, the laboratory, and during fieldwork. Artwork by Dr. Sara Weinstein

A growth mind‐set is demonstrated when someone believes that intelligence/ability can be developed over time. This is contrary to a fixed mind‐set where one believes that intelligence/ability is static. Dr. Carol Dweck and others have conducted considerable research demonstrating the importance of approaching instruction and mentoring with a growth mind‐set (Dewsbury, 2020; Dweck, 1999, 2014; Seaton, 2018). This approach can have tremendous positive impacts on students and mentees, resulting in improved outcomes for traditionally underserved students (Canning et al., 2019). Therefore, as you are reading this paper, we encourage you to have a growth mind‐set—to learn and reflect on your own approaches and practices and be willing to grow and develop a more inclusive framework for your teaching and research (Figure 1).

## 3 BUILDING INCLUSIVITY IN TEACHING AND RESEARCH ENVIRONMENTS

Here, we constrain our discussion to three environments commonly encountered by ecologists and evolutionary scientists: the classroom, the laboratory, and the field. These environments present both shared and unique opportunities and challenges for fostering inclusivity. As you read about these environments, remember that axes of diversity are numerous and not always immediately apparent; it is important to be aware of your own biases and naiveté when working with others.

### 3.1 Teaching in the classroom

Ask yourself: *What barriers to entry am I unknowingly perpetuating in my classroom and through my current teaching practices?*

The classroom has a lasting impact on how students perceive their relationship with science. Along with all of the logistical and skills/content‐based goals and concerns that come with teaching a course, instructor–student interactions can have a significant impact on student success, self‐efficacy (confidence), and science identity (Trujillo & Tanner, 2014). A constructive strategy to guide all of your students to feel and think like scientists is to cultivate an inclusive atmosphere inside and outside of the classroom (Dewsbury, 2020; Dewsbury & Brame, 2019). Some simple practices include facilitating balanced groups, learning names, using pronouns, supportive messaging in your syllabus, and increasing representation and relevance in your teaching materials (Wood et al., 2020). Materials should also be designed with accessibility in mind. An inclusive message is lost if it cannot be perceived.

#### 3.1.1 Balanced groups in the classroom

Group work is a fundamental aspect of working in the sciences, and having students work in groups is known to have numerous benefits for their development and education (Kempa & Ayob, 1995; Seethamraju & Borman, 2009; Thorley & Gregory, 1994). Collaborative learning is an opportunity to increase participation and student–student interactions. In traditional randomly assigned group work, students can feel marginalized or experience increased anxiety (Henning et al., 2019; Juvonen et al., 2019; Rosser, 1998; Strauss et al., 2011). As the instructor, you have the ability to structure groups to be more inclusive and inviting for all students. Engineering groups to balance gender, ethnicity, power structures, and other relevant categories without isolating members of marginalized groups is recommended (Huxham & Land, 2000; Katzenbach & Smith, 1993; Seethamraju & Borman, 2009; Slavin, 1995). While each instructor will have their preference for structuring and assessing groups, there are some strategies available in the literature such as grouping students with similar out‐of‐class schedules, emphasizing flexibility in managing group dynamics (i.e., rotating leaders), and using peer assessment (Clarke & Blissenden, 2013; Hubscher, 2010; Layton et al., 2010; Loughry et al., 2014; Scott, 2017).

#### 3.1.2 Learning student names & using pronouns

Learning student names can help build student–instructor relationships (Tanner, 2011) and create a more positive classroom environment (Tanner, 2013). By simply having name “tents” (folded paper with their name facing the instructor) in the classroom at each student's desk/table and learning to pronounce students’ names correctly, instructors can cultivate a more comfortable environment and build community in the classroom (Cooper et al., 2017; Kohli & Solórzano, 2012). In addition to having names available for reference, including the option for sharing pronouns can also increase transparency and encourage self‐identification (Cooper et al., 2020; Spade, 2011). We suggest providing opportunities for students to self‐identify their pronouns to the instructor discreetly (e.g., through filling out quick surveys on the first day of class), or, if the student is comfortable, with the whole class (Cooper et al., 2020; Pryor, 2015). Modeling this behavior for your students by stating your own pronouns when you introduce yourself to the class sets an example for students and indicates that you take inclusivity seriously. We also acknowledge that learning names and pronouns by traditional methods like name “tents” and photo/name galleries can present barriers to instructors who are blind or low vision, those with print disabilities, and others. Other strategies such as asking students to provide short audio recordings or written bios and establishing the norm of saying one's name before speaking can be useful substitutes.

#### 3.1.3 Inclusive syllabus and establishing norms

In many situations, a syllabus might be the first exposure students have to an instructor and a course. Developing a learner‐focused syllabus (Heim et al., 2019; Palmer et al., 2014) with welcoming language sets the tone for an inclusive learning environment (Harnish & Bridges, 2011; Passman & Green, 2009). This consists of many elements, including a positive and respectful tone, language consistent with a growth mind‐set, encouraging students to explore and ask questions, and recommendations for how students can meet course expectations. Incorporating student feedback into your syllabus can be as simple as providing an online cloud version with student permission to add comments and questions for clarification on course objectives and assignments. Additionally, it is helpful to establish standards for discourse at the beginning of a course; otherwise, noninclusive social norms may guide discourse (Neill et al., 2019). For example, by simply establishing rules around answering questions, raising hands, and debating among students, instructors can reduce male dominance in participation and marginalization of some students (Caspi et al., 2008; Wayne et al., 2010). For more detailed guidance on syllabus construction, we recommend the work by Palmer et al. (2014).

#### 3.1.4 Increasing representation and relevance

Education research shows that social integration, a sense of belonging (Chang et al., 2010; Johnson, 2012; Rainey et al., 2018; Strayhorn, 2018; Walton & Cohen, 2011), and developing a science identity (Hughes & Hurtado, 2013; Trujillo & Tanner, 2014) are important for success and retention of underrepresented groups in STEM. One way to foster a sense of community among students is by increasing the diversity of representation of scientists in the classroom (Egalite et al., 2015; Le & Matias, 2019). By diversifying the scientists that students are exposed to, you can help students identify as scientists and feel like part of the community. Example strategies include highlighting diverse scientists in course topics/material (Schinske et al., 2017; Zemenick & Weber, 2020) and web conferencing with scientists of diverse backgrounds to facilitate interactions between students and professionals. Cultural and community‐relevant materials can also enhance the learning experiences of a diverse student population (Warren et al., 2001). One way to empathize with your students' unique life experiences is by providing space for them to incorporate their experiences into course activities. For example, having open‐ended assessments whereby students have some choice in the direction of their assignment can allow for personalization and the opportunity for students to explore how science affects their daily lives.

### 3.2 Developing an inclusive research laboratory

Ask yourself: *How does the way I manage my research laboratory actively promote diversity and inclusion?*

In ecology and evolutionary research, research groups are often organized into laboratories, whether that means a designated physical space or a grouping of students and researchers under a specific adviser or principle investigator. For undergraduate students, research laboratories may be their first experience with particular cultural norms of scientific inquiry. Therefore, it is extremely important to cultivate a welcoming atmosphere and culture in the laboratory. Fostering an inclusive research laboratory environment requires attention to three broad areas: laboratory member recruitment and selection, interpersonal dynamics, and cultural norms in academic research.

#### 3.2.1 Recruitment and selection of researchers

Bringing students with diverse identities into the research laboratory requires welcoming practices that reflect a diverse scientific community. Students are more interested in research when they feel confident and safe to develop their own scientific identity (Chemers et al., 2011; Riccitelli, 2015). Supporting and encouraging a diversity of students in the research environment begin with recruitment and selection that goes beyond traditional passive strategies such as waiting for email requests or asking laboratory members to suggest candidates.

Active recruitment requires good advertising. Advertisements should be accessible and distributed as multiple media (e.g., physical flyers, web postings, class announcements). The more widely a student research position is advertised, the greater chance it has of being noticed by members of underserved groups. Additionally, depicting many axes of diversity in job advertisements and on laboratory websites shows potential applicants that they are included in the target audience, promoting a sense of belonging even before candidates submit an application (Avery et al., 2004). Even in cases where laboratories may have little visible diversity to depict, statements encouraging students to apply from all backgrounds and experience levels help lower the barrier of perceived exclusion. Inclusive recruitment efforts can go beyond formal advertising. Current laboratory members could discuss their research experiences and its relevance to their life and goals at campus activities and social events to raise awareness about student research and its value and relevance in groups that may not broadly intersect with ecology or evolutionary research communities (Ahmad et al., 2019).

Advertisements should also explicitly address possible misconceptions about work flexibility in research laboratories (Ahmad et al., 2019). Students with outside work or family roles may assume that working hours are not flexible or that remote work is not welcome in research (Fairchild, 2003). Those receiving accommodations for a disability in their courses may believe similar accommodations are not available during the hiring process or in research positions. Students may also have assumptions about academic requirements, grade cutoffs, and test scores. Explicit statements outlining aspects of flexibility, availability of accommodations in the hiring process and the workplace, and academic requirements or lack thereof, lower recruitment barriers and apprehension about who can and cannot do research.

Inclusive candidate selection also involves avoiding implicit biases (Bertrand & Mullainathan, 2004; Eaton et al., 2020). Everyone has them, regardless of intent or identity. Objective evaluation of candidates limits the influence of implicit bias. This means identifying a specific set of skills required to accomplish the goals of the position, criteria for determining whether a candidate possesses each skill, and the relative importance of each skill or trait, before a candidate review begins. Identifying traits that are key to research success, like motivation and curiosity, in addition to specific skills, is also important (Emery et al., 2019). Criteria and evaluation methods can be qualitative while still being objective. The most inclusive evaluation avoids relying solely on criteria that can be biased and are not directly related to the position (e.g., standardized test scores (Berry et al., 2011; Ployhart et al., 2003) and arbitrary grade cutoffs). Instead, evaluation should focus on evidence from multiple sources that relate to the applicant's ability to succeed in the position and avoid the use of extraneous criteria that erect unnecessary barriers to participation.

#### 3.2.2 Interpersonal interactions in research settings

Modeling inclusive behavior as a normal part of social interaction in the laboratory demonstrates empathy and fosters an inclusive atmosphere (Meeussen et al., 2014). Modeling and promoting inclusive behaviors can take many forms, such as providing quality mentorship to postdocs, students, and technicians (Hund et al., 2018; National Academies of Sciences, Engineering, & Medicine, 2019) or initiating open dialogue surrounding systemic racism in academia (Chaudhary & Berhe, 2020; Gewin, 2020). Mentors who openly acknowledge and celebrate diversity rather than taking a diversity‐blind approach to research mentorship will have more inclusive and productive labs (Campbell et al., 2013; Morales et al., 2017; Page, 2008). Actively engaging in and creating space for discussion of issues related to diversity, equity, and inclusion (e.g., at group meetings) can increase laboratory members' comfort in openly discussing such topics (Sabat et al., 2017). Choosing to participate in campus efforts aimed at increasing diversity and inclusion and attending diversity‐related trainings and events demonstrates to laboratory members that these actions are appropriate and valuable uses of their time. These behaviors also demonstrate a growth mind‐set in an advisor's approach to their own laboratory culture, showing that inclusivity is an ongoing, iterative process.

Inherent power imbalances among PIs, graduate students, postdocs, staff scientists, and undergraduate researchers make establishing social norms in the laboratory critical. All laboratory members should know what constitutes acceptable and unacceptable behavior. They also need to know what to do and who to contact if they feel those expectations are being violated. An effective code of conduct addresses these needs (Nitsch et al., 2005; see laboratory group code of conduct examples in Appendix S1D). Ideally, one of the individuals listed as a contact person or ombudsperson should not be reliant on the laboratory's PI for employment or future career success to reduce the influence of power dynamics when resolving conflicts. An explicit description of social norms to which all laboratory members agree promotes a safe, inclusive environment for all members, regardless of position.

#### 3.2.3 Research and academic cultural norms

Every research laboratory has its own “ways of doing things,” and research approaches in ecology and evolution each have their own best practices. Some of these structures, like specific protocols, may be explicit, while others, like use of common spaces, are implicit. Similarly, some criteria for research success as measured by graduate programs, scholarship/fellowship applications, grants, and job applications, are explicit while others are implicit.

Having a centralized virtual or physical location for laboratory procedures and protocols along with a standardized onboarding process for all new laboratory members is one way to make laboratory procedures explicit and, therefore, more inclusive. Members can be given a written, recorded, and/or, ideally, real‐world walk‐through of common laboratory practices relevant to their position. This could include topics such as waste disposal, cleaning equipment, replacing stock solutions, data storage and access, shared computational resources, and miscellaneous practices every laboratory member is just “expected to know.” Providing this information at the onset creates an atmosphere where no one has a monopoly on key information. An onboarding process also provides an ideal opportunity to introduce the code of conduct discussed above.

Mentors who embrace a growth mind‐set can guide students through the nuanced expectations for professional materials such as applications, personal statements, and cover letters. This puts all members, especially those from historically underserved groups, in a more competitive position for career advancement (Mathur et al., 2019; McKay & Davis, 2008; Sedlacek, 2017). Working with individuals to establish research goals and paths to achievement recognizes laboratory members' unique backgrounds and reduces barriers for those who are less familiar with research and academic norms. Tools like Individual Development Plans (Tsai et al., 2018) and student contracts (Emery et al., 2019) can help with transparency and communication between mentor and mentee.

### 3.3 Making the field welcoming to all

Ask yourself: *How might implicit biases, systems of oppression, and power dynamics affect my interactions with scientists and/or students while in the field?*

As ecologists and evolutionary biologists, the questions we pursue often involve conducting fieldwork at some point in our careers. Fieldwork can present unique challenges, such as ensuring that students and employees have access to field experiences and that they feel safe and supported during those experiences. Making field experiences inclusive and welcoming for everyone requires advanced preparation on multiple fronts, including in hiring practices, discussing facilities and responsibilities in the field, creating a field‐specific code of conduct to establish and sustain behavioral norms, and addressing accessibility in the field.

#### 3.3.1 Advanced preparation for fieldwork

Facilitating safe and supportive fieldwork for everyone starts well before entering the field. First, as mentioned in the previous section on building an inclusive laboratory environment, implicit biases can often influence the hiring process (Bertrand & Mullainathan, 2004; Eaton et al., 2020). To make fieldwork accessible to all, the same strategies for recruitment, selection, and retention of laboratory members also apply when engaging with students and technicians who will be conducting fieldwork.

Fieldwork comes in many forms, and having open and clear conversations about field conditions and expectations is key to successful and safe working conditions. In more formal educational contexts where classes have fieldwork components, you will likely be interacting with students who have varying levels of experience with fieldwork; some students may be regaling friends and classmates with stories from “last summer at field camp,” while others might feel uncertain about what the term “fieldwork” entails (Giles et al., 2020; Núñez et al., 2019). There might be similar discrepancies in experiences when hiring technicians or graduate students (Fournier & Bond, 2015). As a mentor, it is important to acknowledge that mentees do not need previous outdoor experience to be capable and enthusiastic field researchers. Regardless of the amount of previous field experience, fieldwork can introduce unique challenges, including: reduced independence in terms of access to transportation, food, facilities, and medical/mental health resources; unfamiliar cultural practices or norms; distance from support networks; long days with physically strenuous activity; and greater exposure to potentially unfamiliar environmental hazards (John & Khan, 2018). Additionally, scientists of color—especially Black scientists—are likely acutely aware that they may face unwarranted discrimination or violence in outdoor spaces, particularly in the United States (Blahna & Black, 1992; Demery & Pipkin, 2020; Goodrid, 2018; West, 1989). Similarly, travel can be difficult or dangerous for students or employees for a number of reasons, such as anti‐LGTBQ + laws and visa/documentation limitations (Prior‐Jones et al., 2020). Any or all of these aspects may generate discomfort or concern; such feelings should be met with empathy and active discussion about how best to mitigate these concerns rather than ignored, brushed aside, or ridiculed. Discussing field conditions and expectations beforehand gives everyone a chance to mentally acclimate to the new situation, ask clarifying questions, and have time to prepare appropriately (John & Khan, 2018; Starkweather et al., 2018).

#### 3.3.2 Field‐specific codes of conduct

As previously mentioned, establishing a laboratory code of conduct is important for creating a safe and secure social environment in a research group. Fieldwork adds the additional complexity of taking place in novel and/or remote locations, where a perceived (and often real) lack of accountability and enforcement can increase the probability of hazing, physical or verbal intimidation, and sexual harassment (Clancy et al., 2014; Nelson et al., 2017). Therefore, if you manage a research group that conducts fieldwork, we encourage the creation of a field‐specific code of conduct that reduces any ambiguity about behavioral norms. This can (and likely will) be similar to your research group's code of conduct or even a subsection of the laboratory code of conduct; something similar can be put into effect for classes which have fieldwork components. For examples of fieldwork codes of conduct, see Appendix S1D. Be clear that the same rules of safety and respect that students or laboratory members agree to abide by within the laboratory or classroom also apply when in the field. Additionally, clear reporting guidelines should be put into place (Nitsch et al., 2005); while these may mirror those of the laboratory, different guidelines may be required based on who will be in the field and which methods of communication will be available.

#### 3.3.3 Awareness of cost barriers in field research

The cost of gear is also a potential barrier to fieldwork and is often overlooked (Núñez et al., 2019). Unlike working in an office or laboratory setting, experiences that include fieldwork often require participants—students and employees alike—to provide at least some of their own gear; this can be in the form of attire (e.g., hiking boots, field pants), general supplies (e.g., water bottles, backpacks), more extensive gear (e.g., tents, sleeping bags) (Giles et al., 2020; Ham & Flood, 2009), or personal vehicles for transportation to field sites. When grades are determined by whether students are wearing the correct gear for a field trip, this can have a disproportionately negative effect on students who are financially insecure (Giles et al., 2020; Ham & Flood, 2009; Walpole, 2003). Approach these issues with empathy and flexibility by making conscientious decisions about what gear is in fact “required.” For example, if tennis shoes or closed‐toed shoes will suffice in place of hiking boots, there is no need to make hiking boots a requirement. Additionally, if at all possible, have extras of necessary supplies on hand for students who cannot afford them or help facilitate a gear swap or other borrowing system (Giles et al., 2020).

#### 3.3.4 Accessibility in the field

When designing a class with a field trip or fieldwork, a flexible design to embrace the broadest diversity of students is the best strategy. In higher education in the United States, for example, legal responsibility for requesting specific accommodations on the basis of disability is placed on students (Hadley, 2011). As such, many instructors find out about needed accommodations on the first day of class or, in some cases, may never be made aware (Feig et al., 2019). Students may not disclose their disability for a number of reasons, including not being aware of their own disability, social stigma, or delays in approval from the institutions (Cole & Cawthon, 2015; De Cesarei, 2015). Making last minute accommodations for a trip can be challenging and frustrating for all involved and can lead to students with disabilities being excluded from participation (Feig et al., 2019). For field trips or fieldwork, we recommend not making assumptions about a person's comfort level or abilities. Preemptively designing activities with the flexibility to transition between modes of instruction and meet the needs of the broadest diversity of abilities and backgrounds increases inclusivity; it not only reduces the likelihood that students with disabilities will be excluded but also benefits other students, with or without disabilities (Feig et al., 2019).

A genuine and sincere effort should be made to allow all participants to be involved, though we acknowledge that it is sometimes impossible to make every aspect of field activity accessible to everyone. For example, if your research *requires* off‐trail, backcountry hiking to remote locations, you may not be able to make all aspects of the project accessible to someone who has severely limited mobility (depending on the specific environment and precise nature of mobility limitation). Nevertheless, difficulty or inability to make fieldwork accessible to everyone should not be an excuse to ignore accessibility issues and simply delegate tasks to a person for whom participation is achievable (Carabajal et al., 2017). If—after brainstorming, discussion, and genuine attempts at making accommodations—all parties are in agreement that sufficient accommodations cannot be made for a particular task or experience, then a student or employee can work on another aspect of the project that provides a path to achieving the similar learning goals or job objectives (Carabajal et al., 2017). These recommendations are not meant to be legal advice, but rather humane advice. Guidelines for each institution may vary, so for advice on legal compliance (as well as suggestions for how you can meet student needs), we recommend working with your institution's Office of Accessible Education or equivalent.

While we recommend making fieldwork as accessible as possible to those who wish to participate, we also want to be clear that conducting fieldwork is not a requisite for success in ecology or evolutionary science. There are many paths to being an ecologist, evolutionary biologist, and not all of them include field experience, especially given the long history of excellent research work conducted in laboratory settings and growing trend toward big data and computational work (Giles et al., 2020; Peters et al., 2014). Fieldwork should not be subject to ability gatekeeping (Feig et al., 2019), nor should fieldwork be used as a gatekeeper to becoming an ecologist or evolutionary biologist (Giles et al., 2020).

## 4 CONCLUSION

As researchers and instructors in ecology and evolutionary science, we often need to adapt and change our approaches to scientific inquiry. We advocate that scientists leverage these skills to take an inclusive approach in their research and teaching, providing a welcome scientific and learning environment for everyone. By exercising empathy toward others, embedding flexibility into structures, and practicing a growth mind‐set as part of a dedicated journey in self‐reflection, scientists can build a more inclusive environment in any setting. Whether in a classroom, the research laboratory, or the field, scientists can make educated choices about how they structure these environments and conduct themselves to better include people of all identities and backgrounds. This provides space for yourself, students, and mentees to bring their whole selves into the classroom and research with ready‐made validation. When you take the time to connect with students and mentees and invest in improving and reflecting on your practices, in small steps or big, you contribute to building a diverse and intellectually engaged community in ecology and evolutionary science.

## CONFLICT OF INTEREST

None declared.

## AUTHOR CONTRIBUTION

**Nathan C. Emery:** Conceptualization (lead); Resources (supporting); Writing‐original draft (lead); Writing‐review & editing (lead). **Ellen K. Bledsoe:** Conceptualization (equal); Resources (lead); Writing‐original draft (equal); Writing‐review & editing (supporting). **Andrew O. Hasley:** Conceptualization (supporting); Resources (supporting); Writing‐original draft (supporting); Writing‐review & editing (supporting). **Carrie Diaz Eaton:** Conceptualization (supporting); Supervision (equal); Writing‐review & editing (equal).

## Supporting information

Appendix S1

## Data Availability

There are no data associated with this article.
