# Supplementary material for: Cultivating inclusive instructional and research environments in ecology and evolutionary science
Source: Ecol Evol. 2020 Dec 7;11(4):1480–91. doi: 10.1002/ece3.7062 (PMC7882980; doi:10.1002/ece3.7062)
Supplement: Supplementary file 1 — Appendix S1 [file ECE3-11-1480-s001.pdf]

Cultivating inclusive instructional and research environments in ecology and evolutionary science

Appendix 1

A. Allyship

In our paper, we primarily identify ways in which educators and scientists from any background can make the classroom, the lab, and the field more inclusive, welcoming spaces. Another way to continue working towards making science more inclusive is by being allies to marginalized, oppressed, or underserved groups. As Kim Case says in her 2013 book on allies in the classroom, an ally is "a dominant group member 'who works to end oppression in [their] personal or professional life through support of, and as an advocate with and for, the oppressed population'" (Washington & Evans, 1991 *in* Case, 2013). Allyship focuses specifically on how individuals can leverage their privilege or power in a given situation to make meaningful change. Because privilege (and, conversely, oppression) is intersectional—meaning that combinations of one's social, cultural, and political identities create unique modes of power and discrimination (Crenshaw, 1989)—*we all have the ability to be allies in different situations*. For example, a Black cisgender man can be an ally to a white transgender, non-binary person if someone makes a transphobic remark; on the other hand, a white non-binary person can be an ally to a Black man if someone makes a racial microaggression. While it is fairly common and accepted for non-members of the LGBTQ+ community to self-identify as an ally, in most other instances, self-declaration or self-nomination of allyship is not encouraged. We recommend using your actions to demonstrate allyship rather than calling yourself an ally. Active allyship can take many forms; it can be in response to a specific incident, and it can also be pre-emptive by actively creating a supportive environment. Unequivocally, however, being an ally to an oppressed group requires 1) understanding your own privilege and 2) *intentional* and *active* work to end oppression and to promote an inclusive environment.

Quick Allyship Tips:

- Self-educate to understand your own privilege and power. Do NOT ask a member of a marginalized group to educate you on systems of oppression. (e.g. A list of anti-racism resources available here: https://inclusion.msu.edu/education/Resources.html)
- Do, however, ask how you can best support someone; people like to be supported in different ways.
- Remember that retaliation is more likely to occur against members of oppressed groups than allies who speak up about oppression. Do not put a member of a marginalized group in harm's way without consent.
- The onus to respond to an incident of aggression against an oppressed group should not be on a member of the oppressed group; being an ally means taking the burden of response upon yourself. However, if a member of the oppressed group chooses to address the incident, step back and support them as best you can. Do not take over the situation unless asked or specifically given permission.

- Quick responses to rapidly de-escalate the situation are fine (e.g., “Yikes!” or “We don’t say that here.”). If needed, you can follow up with the offender at a later point to discuss the nuances of the situation. As an educator in a classroom, however, an explanation of the issue and addressing the harm caused can be a powerful teaching moment.
- Do not target other marginalized or oppressed groups while defusing the situation. Humor often backfires.
- Be willing to make mistakes and learn from them. Thank the person who pointed out the mistake, apologize, and commit to doing better. Be sure not to create a situation where the other person has to make you feel better about your error.

For additional information on allyship and how to use your privilege to fight oppression and promote inclusive environments, we encourage you to visit the “Dear Ally Skills Teacher” blog at https://dearally.com/. For addressing privilege in the classroom, we recommend *Deconstructing Privilege: Teaching and Learning as Allies in the Classroom* by Kim Case (2013).

B. Organizations/Websites of Interest

Environmental Data Science Inclusion Network (EDSIN) - A network dedicated to strengthening and collaborating across initiatives to recruit and retain individuals from underrepresented groups in data science careers. <https://qubeshub.org/community/groups/edsin/>

Biological, Universal, and Inclusive Learning in Data Science (BuiLDS) - A community for the exchange and sharing of resources supporting biological and environmental data science education, grounded in universal design and inclusive pedagogy. <https://qubeshub.org/community/groups/builds>

NSF INCLUDES National Network - A coordination hub for building a national movement to broaden participation in STEM <https://www.includesnetwork.org/new-a/coordinationhub>

ADVANCEGeo Partnership - An organization for empowering the geosciences community (and other STEM+ fields) to transform the workplace climate. Resources include guides for writing codes of conduct, making fieldwork more inclusive, etc. <https://serc.carleton.edu/advancegeo/resources/index.html>

Guide to Inclusive Scientific Meetings - The 500 Women Scientists organization has compiled a thorough guide to organizing inclusive scientific meetings. <https://500womenscientists.org/inclusive-scientific-meetings>

C. Recommended further reading/viewing in Universal Design for Learning (UDL)

A short video describing UDL and why it is useful: <https://www.youtube.com/watch?v=GcJ1eJQar8o>

A comprehensive overview of Universal Design for Learning:
Meyer, A., Rose, D.H., & Gordon, D. (2014). Universal design for learning: Theory and Practice. Wakefield, MA: CAST Professional Publishing. <http://www.cast.org/our-work/publications/2014/universal-design-learning-theory-practice-udl-meyer.html>

Guidance for adopting, implementing, and advocating for UDL in higher education, emphasizing that UDL is not just for students with disabilities:
Tobin, T. J., & Behling, K. T. (2018). Reach everyone, teach everyone: Universal design for learning in higher education. West Virginia University Press.

An easily digestible table of the UDL guidelines with links to definitions, examples, and supporting research: <http://udlguidelines.cast.org/>

D. Code of Conduct Examples:

Lab:

- Poisot Lab <https://poisotlab.io/values/>
- Weecology Lab <https://www.weecology.org/lab-wiki/code-of-conduct/>
- Sedimentary Basins Research Group <https://risingfluids.com/coc/>

Field:

- See ADVANCEGeo Code of Conduct resources
- See Appendix F of UW's Preventing Harassment in Fieldwork Situations: Report from the University of Washington's Respect and Equality in Fieldwork (REIF) 2017 Committee: <http://psc.apl.washington.edu/HLD/REIF/RespectandEqualityinFieldwork_RecommendationsandReportUW_Jan2018.pdf>
- Google doc from Dr. Sarah Perry, Director of Research & Engagement at the Museum of London Archaeology (MOLA): <https://docs.google.com/document/d/1WhbeE4dWI_FagMlU6ZUX9_tUpou5OAsg-FQtSABWQ2w/>

E. Reference

Washington, J., & Evans, N. J. (1991). 'Becoming an ally.' In N. J. Evans, & V. Wall (Eds.) Beyond tolerance: Gays, lesbians and bisexuals on campus. Lanham, MD, USA: American College Personnel Association.
